# Determinants of prolonged exclusive breastfeeding among children aged 6–23 months in 21 sub-saharan African countries: evidence from nationally representative data

**DOI:** 10.1186/s13052-025-01856-5

**Published:** 2025-01-23

**Authors:** Enyew Getaneh Mekonen

**Affiliations:** Department of Surgical Nursing, School of Nursing, College of Medicine and Health Sciences, University of Gondar, Gondar, Ethiopia

**Keywords:** Prolonged exclusive breastfeeding, Children, Sub-saharan Africa, DHS, Multilevel analysis

## Abstract

**Background:**

Under-five mortality and malnutrition are more common in many low- and middle-income countries, highlighting the grave consequences of improper nutrition for children. Infants that continue to be exclusively breastfed after six months are considered to be engaging in prolonged exclusive breastfeeding. Children with prolonged exclusive breastfeeding are more susceptible to anemia, atopic dermatitis, and food allergies. There is no evidence on the pooled prevalence and determinants of prolonged exclusive breastfeeding in sub-Saharan Africa. Therefore, this study is intended to determine the prevalence and associated factors of prolonged exclusive breastfeeding among children aged 6 to 23 months in sub-Saharan African countries.

**Methods:**

Data from the recent demographic and health surveys of 21 countries in sub-Saharan Africa conducted between 2015 and 2022 were used. A total weighted sample of 63,172 mother-child pairs was included in the current study. Multilevel mixed-effects logistic regression was used to determine the factors associated with the outcome variable. Intra-class correlation coefficient, likelihood ratio test, median odds ratio, and deviance (-2LLR) values were used for model comparison and fitness. Finally, variables with a p-value < 0.05 and an adjusted odds ratio with a 95% confidence interval were declared statistically significant.

**Results:**

The pooled prevalence of prolonged exclusive breastfeeding among children aged 6–23 months in sub-Saharan Africa was 17.32% (95% CI: 17.03%, 17.62%). Factors like child age [AOR = 4.39; 95% CI (4.17, 4.62)], wealth index [AOR = 1.15; 95% CI (1.07, 1.23)], maternal educational level [AOR = 1.56; 95% CI (1.36, 1.78)], marital status of the mother [AOR = 1.11; 95% CI (1.04, 1.19)], media exposure [AOR = 1.11; 95% CI (1.06, 1.17)], place of delivery [AOR = 0.82; 95% CI (0.78, 0.87)], postnatal checkup [AOR = 1.43; 95% CI (1.36, 1.51)], drinking water source [AOR = 1.06; 95% CI (1.01, 1.11)], sanitation facility [AOR = 1.15; 95% CI (1.10, 1.21)], antenatal care attendance [AOR = 1.27; 95% CI (1.16, 1.39)], community literacy [AOR = 1.08; 95% CI (1.02, 1.15)], and community media exposure [AOR = 1.06; 95% CI (1.01, 1.13)] were significantly associated with prolonged exclusive breastfeeding.

**Conclusions:**

Nearly one out of five children aged 6–23 months in sub-Saharan Africa had prolonged exclusive breastfeeding. Both individual- and community-level factors were significantly associated with prolonged exclusive breastfeeding. Policymakers could find it very important to support maternal education, poverty reduction, media exposure, maternal healthcare services, and complementary feeding hygiene practices in order to encourage the timely initiation of complementary feeding.

## Background

A healthy diet is essential for children’s normal growth and development. During the first two years of life, there is a crucial window of opportunity to promote optimal growth and development if proper child-feeding practices are followed [1]. Early breastfeeding initiation, exclusive breastfeeding, timely and safe introduction of supplemental feeding, and continuous breastfeeding until the child turns two years old or older are all components of infant and young child feeding practices that are crucial during the first two years of life [2]. Appropriate complementary feeding practices have been estimated to save 6% of under-five mortality annually and to lead to a 17% reduction in the prevalence of stunting at 24 months of age [3, 4]. The primary causes of undernutrition in the first two years of life are infectious diseases and inadequate breastfeeding practices [5].

The World Health Organization (WHO) advises that the infant be given just breastmilk until the child is six months old [6]. This will improve the child’s health and survival rate throughout this time and into adulthood [7]. In addition to providing protective qualities against disease, maternal milk provides the right amount of energy and nutrients for the infant’s physiological maturity, making it perfect for the first few months of life [5, 8]. When the amount and content of mother milk are no longer adequate to meet the infant’s nutritional needs, it is crucial to start supplemental feeding after six months of age [9].

For the infant to grow and develop as best it can, solid, semi-solid, or soft foods must be introduced (complementary feeding). This is especially important after the first six months of life, when breast milk is no longer enough to meet the infant’s nutritional and developmental needs [10]. Adverse child health outcomes are linked to the inappropriate introduction of supplemental foods, which can have long-term repercussions [11, 12]. In infants aged 6 to 8 months, a delayed introduction of complementary foods has been linked to stunting and severe stunting [13]. Under-five mortality and malnutrition (i.e., stunting, wasting, and underweight) are more common in many low- and middle-income countries (LMICs), highlighting the grave consequences of improper nutrition for children [14].

In the first six to eight months of life, untimely supplementation with complementary foods might increase the risk of macro- and micronutrient deficiencies in newborns and young children, which can stunt their growth [15]. Furthermore, supplementary foods become a significant source of iron and other nutrients required for hemoglobin synthesis after six months of life, meeting the majority of a child’s iron needs between the ages of 12 and 23 months [16]. Infants who do not receive complementary foods in a timely manner may be at higher risk of developing iron deficiency anemia. Over a six-month period, infants solely breastfed had considerably lower blood iron and ferritin levels than those who were supplemented with complementary foods [17]. The necessity to concentrate on food-based indicators of a child’s nutritional status is further implied by the possibility that the late introduction of complementary foods may even affect immune and brain development, with short- and long-term effects [18, 19].

Infants that continue to be exclusively breastfed after six months are considered to be engaging in prolonged exclusive breastfeeding (PEB), which goes against the guidelines for feeding infants and young children and may deprive the child of nutrients essential for healthy growth and development [20]. According to earlier research, children with PEB may be more susceptible to atopic dermatitis and food allergies [21]. Likewise, a higher risk of anemia in newborns aged 12 months and lower hemoglobin concentrations in both infants and young children aged 48–71 months were linked to exclusive breastfeeding beyond 6 months [22]. As far as the researcher’s knowledge is concerned, there is no evidence on the pooled prevalence and determinants of PEB in sub-Saharan Africa (SSA). Therefore, this study is intended to determine the prevalence and associated factors of PEB among children aged 6 to 23 months in SSA countries using the recent demographic and health survey (DHS) data.

## Methods and materials

### Data source, study design, and sampling

Using the most recent DHS data from 21 SSA nations, collected between 2015 and 2022, a cross-sectional pooled dataset was used. Angola (2015-16), Benin (2017-18), Burundi (2016-17), Ethiopia (2016), Gabon (2019-21), Ghana (2022), Gambia (2019-20), Guinea (2018), Kenya (2022), Liberia (2019-20), Mali (2018), Malawi (2015-16), Nigeria (2018), Rwanda (2019-20), Sierra Leone (2019), Senegal (2019), Tanzania (2022), Uganda (2016), South Africa (2016), Zambia (2018), and Zimbabwe (2015) were among the 21 SSA countries whose demographic and health surveys were used. The data were appended to determine the pooled prevalence of PEB in SSA and identify the factors associated with it. Each country’s survey has different datasets, such as those for males, females, children, births, and households. The kid’s record (KR) file was employed in this investigation. The DHS is a national survey that is primarily conducted in LMICs every five years. By using common methods for sampling, questionnaires, data collection, cleaning, coding, and analysis, it enables cross-national comparison [23]. A total weighted sample of 63,172 children aged 6 to 23 months who are living with their mother were included in the present study (Table 1). The DHS uses a two-stage, stratified sampling method [24]. The first step is creating a sample frame, which is a list of enumeration areas (EAs) or primary sampling units (PSUs) that encompass the entire nation. This list is typically created using the most recent national census that is available. The systematic sampling of the households included in each cluster, or EA, is the second step. More details on survey sample techniques are available in the DHS guidelines [25].

**Table 1:** Sample size for prolonged exclusive breastfeeding and its associated factors among children aged 6–23 months in sub-saharan African countries

Country	Year of survey	Weighted sample (*n*)	Weighted sample (%)
Angola	2015-16	4,009	6.35
Benin	2017-18	3,884	6.15
Burundi	2016-17	3,858	6.11
Ethiopia	2016	2,822	4.47
Gabon	2019-21	1,741	2.76
Ghana	2022	2,786	4.41
Gambia	2019-20	2,305	3.65
Guinea	2018	1,909	3.02
Kenya	2022	2,753	4.36
Liberia	2019-20	1,523	2.41
Mali	2018	2,713	4.29
Malawi	2015-16	4,747	7.51
Nigeria	2018	8,883	14.06
Rwanda	2019-20	2,297	3.64
Sierra Leone	2019	2,643	4.18
Senegal	2019	1,770	2.80
Tanzania	2022	3,079	4.87
Uganda	2016	4,160	6.59
South Africa	2016	877	1.39
Zambia	2018	2,785	4.41
Zimbabwe	2015	1,628	2.58
Total sample size		63,172	100

### Variables of the study

#### Outcome variable

According to the WHO, exclusive breastfeeding is defined as feeding exclusively on breast milk or expressed breast milk and avoiding any other liquids or solids, with the exception of drops or syrups containing vitamin or mineral supplements or medications [20]. The study’s outcome variable was prolonged exclusive breastfeeding (no, yes), which is defined as a child’s exclusive breastfeeding intake between the ages of 6 and 23 months [22].

#### Explanatory variables

The current study took into account both the individual and community levels in order to accommodate the hierarchical nature of DHS data. At the individual level, factors like child age (6–8 months, 9–11 months, 12–23 months), child sex (male, female), household wealth (poor, middle, rich), maternal education (no education, primary, secondary, higher), maternal age (15–24 years, 25–34 years, 35–49 years), marital status (unmarried, married), media exposure (no, yes), place of delivery (home, health facility), postnatal checkup (no, yes), breastfeeding initiation (≥ 1 h of birth, < 1 h of birth), drinking water source (unimproved, improved), sanitation facility (unimproved, improved), and antenatal care visits (< 4, 4–7, ≥ 8) were included. Community-level factors: place of residence (urban, rural), community literacy (low, high), community-level poverty (low, high), and community media exposure (low, high).

### Description of explanatory variables

#### Household wealth

Categorized to three by combining poorest and poorer into one category, “poor,” middle wealth level into the second category, “middle,” and richer and richest into the third category, “rich.”

#### Drinking water source

Improved (use of piped water into dwelling, piped water to yard/plot, public tap/standpipe, tube well or borehole, protected well, protected spring, and rainwater collection) and unimproved (use of unprotected wells, unprotected springs, surface water (river, dam, lake/ponds/stream/canal/irrigation channel), tanker trucks, and carts with small tanks) [26].

#### Sanitation facility

Improved (flush or pour-flush to piped sewer system, septic tank or pit latrine, ventilated improved pit latrine, pit latrine with slab, and composting toilet) and unimproved (flush or pour-flush to elsewhere, pit latrine without slab or open pit, bucket, hanging toilet or hanging latrine, and no facilities or bush or field (open defecation) [26].

#### Media exposure

A variable that is coded as “yes” if the mother was exposed to at least one of these media and “no” otherwise. It is generated by combining the respondent’s preferences for reading newspapers or magazines, listening to the radio, and watching television.

#### Community media exposure

The percentage of women who have been exposed to at least one media outlet, such as a newspaper, radio, or television. It is classified as low (communities where ≤ 50% of women are exposed) or high (communities where > 50% of women are exposed) based on the national median figure.

#### Community literacy

The percentage of women who have completed at least primary school, as determined by survey respondents’ educational attainment. Next, it was divided into two groups based on the national median value: low (communities where ≤ 50% of women have completed primary school) and high (communities where > 50% of women have completed primary education).

#### Community poverty level

This was recoded as low and high community poverty level based on an aggregated variable from household wealth status: low (communities where ≤ 50% of women were poor) and high (communities where > 50% of women were poor).

### Data management and analysis

STATA/SE version 14.0 statistical software was used to clean, recode, and analyze data that was taken from the most recent DHS data sets. To control for non-responses and sampling errors, a sample weight was used. After categorizing continuous variables, categorical variables underwent additional reclassification. The results were presented in frequencies and percentages using descriptive analysis. Descriptive statistical methods were used to portray the variables at the individual and community levels. The variables in the DHS data were arranged into clusters; households were nested within 1692 clusters, and 63,172 children are nested inside households. In order to use the conventional logistic regression model, the presumptions of independent observations and equal variance across clusters were broken. This suggests that accounting for between-cluster effects requires the use of a complex model. Multilevel mixed-effects logistic regression was therefore employed to identify the variables associated with PEB. The null model (outcome variable only), model I (only individual-level variables), model II (only community-level variables), and model III (both individual and community-level variables) are the four models that multilevel mixed effect logistic regression uses. The null model, which is devoid of independent variables, was employed to examine the variation in PEB within the cluster. Evaluations were conducted on the relationships between the outcome variable (Model I) and the factors at the individual and community levels (Model II). The link between the community- and individual-level variables and the outcome variable was fitted simultaneously in the final model (Model III). Through the use of the intra-class correlation coefficient (ICC) and proportional change in variance (PCV), the magnitude of the clustering effect and the extent to which community-level factors explain the unexplained variance of the null model were assessed. The best-fitting model was determined to be the one with the lowest deviance. Ultimately, factors were deemed statistically significant when they had a p-value of less than 0.05 and an adjusted odds ratio (AOR) with a 95% confidence interval (CI) associated with PEB.

### Random-effect analysis results

The methods of estimating random effects or measures of variation of the outcome variable were the PCV, ICC, and median odds ratio (MOR). The variation between clusters was measured by the ICC and PCV. Taking clusters as a random variable, the ICC reveals that the variation of PEB between clusters is computed as ICC = VC/(VC + 3.29) ×100%. The MOR is the median value of the odds ratio between the area of the highest risk and the area of the lowest risk for PEB when two clusters are randomly selected using clusters as a random variable; MOR = 𝑒 ^0.95√VC^. In addition, the PCV demonstrates the variation in the prevalence of PEB explained by factors and computed as PCV = (Vnull-VC)/Vnull×100%, where Vnull = variance of the null model and VC = cluster level variance [27]. The fixed effects were used to estimate the association between the likelihood of PEB and individual and community-level independent variables.

## Results

### Individual- and community-level characteristics of study subjects

A total of 63,172 study subjects were included in the current study. The mean age of children was 14.20 ± 0.02 months, and 64.98% of them fall in the age range of 12–23 months. Regarding child sex, 50.87% of the children were male. Only 4.39% of mothers completed higher education, and 48.10% of them had poor household wealth status. The mean age of mothers was 28.15 ± 0.03 years, and 46.04% of them fall in the age range of 25–34 years. The majority (86.24%) of mothers were married, and 63.80% of them had media exposure. Nearly one-third (33.83%) of mothers had a postnatal checkup, and 26.04% of them gave birth at home. Only 8.43% of mothers had eight or more ANC visits during their recent pregnancy, and 62.32% of them initiated breastfeeding within one hour of birth. More than half (52.91%) of the mothers had unimproved sanitation facilities, and 63.19% of them had improved drinking water sources. More than two-thirds (68.23%) of the mothers were from rural areas, and 55.90% of them were from communities with low levels of literacy. More than half (52.41%) and 53.72% were from communities with high levels of poverty and low levels of media exposure, respectively (Table 2).

**Table 2:** Individual- and community-level characteristics of study subjects, pooled data from 21 SSA countries, DHS 2015–2022

Variables	Category	Frequency (*n*)	Percentage (%)
Child age	6–8 months	11,434	18.10
	9–11 months	10,687	16.92
	12–23 months	41,051	64.98
Child sex	Male	32,135	50.87
	Female	31,037	49.13
Household wealth	Poor	30,390	48.10
	Middle	12,531	19.84
	Rich	20,251	32.06
Maternal education	No education	22,334	35.35
	Primary	20,848	33.00
	Secondary	17,219	27.26
	Higher	2,771	4.39
Maternal age	15–24 years	21,307	33.73
	25–34 years	29,083	46.04
	35–49 years	12,782	20.23
Marital status	Unmarried	8,692	13.76
	Married	54,480	86.24
Media exposure	No	22,871	36.20
	Yes	40,301	63.80
Place of delivery	Home	16,447	26.04
	Health facility	46,725	73.96
Postnatal checkup	No	41,597	66.17
	Yes	21,269	33.83
Breastfeeding initiation	≥ 1 h of birth	23,804	37.68
	< 1 h of birth	39,368	62.32
Drinking water source	Unimproved	23,253	36.81
	Improved	39,919	63.19
Sanitation facility	Unimproved	33,422	52.91
	Improved	29,750	47.09
Antenatal care visits	< 4	39,588	62.85
	4–7	18,089	28.72
	≥ 8	5,311	8.43
Place of residence	Urban	20,068	31.77
	Rural	43,104	68.23
Community literacy	Low	35,310	55.90
	High	27,862	44.10
Community poverty level	Low	30,062	47.59
	High	33,110	52.41
Community media exposure	Low	33,938	53.72
	High	29,234	46.28

### Pooled prevalence of prolonged exclusive breastfeeding

**Fig. 1:**
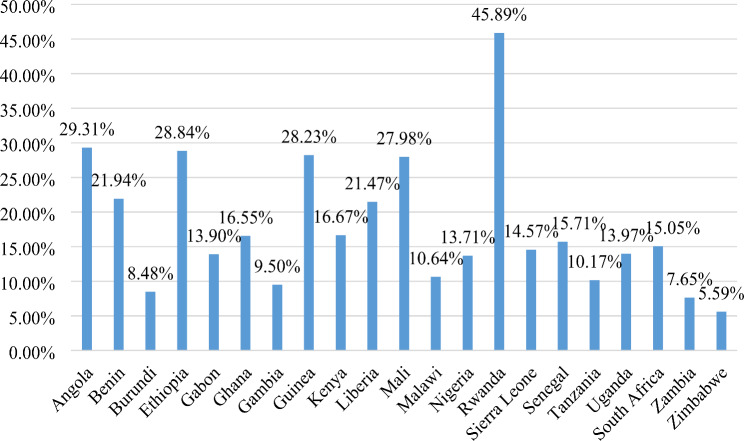
Prevalence of prolonged exclusive breastfeeding by country among children aged 6–23 months in sub-Saharan African countries, DHS 2015–2022

**Fig. 2:**
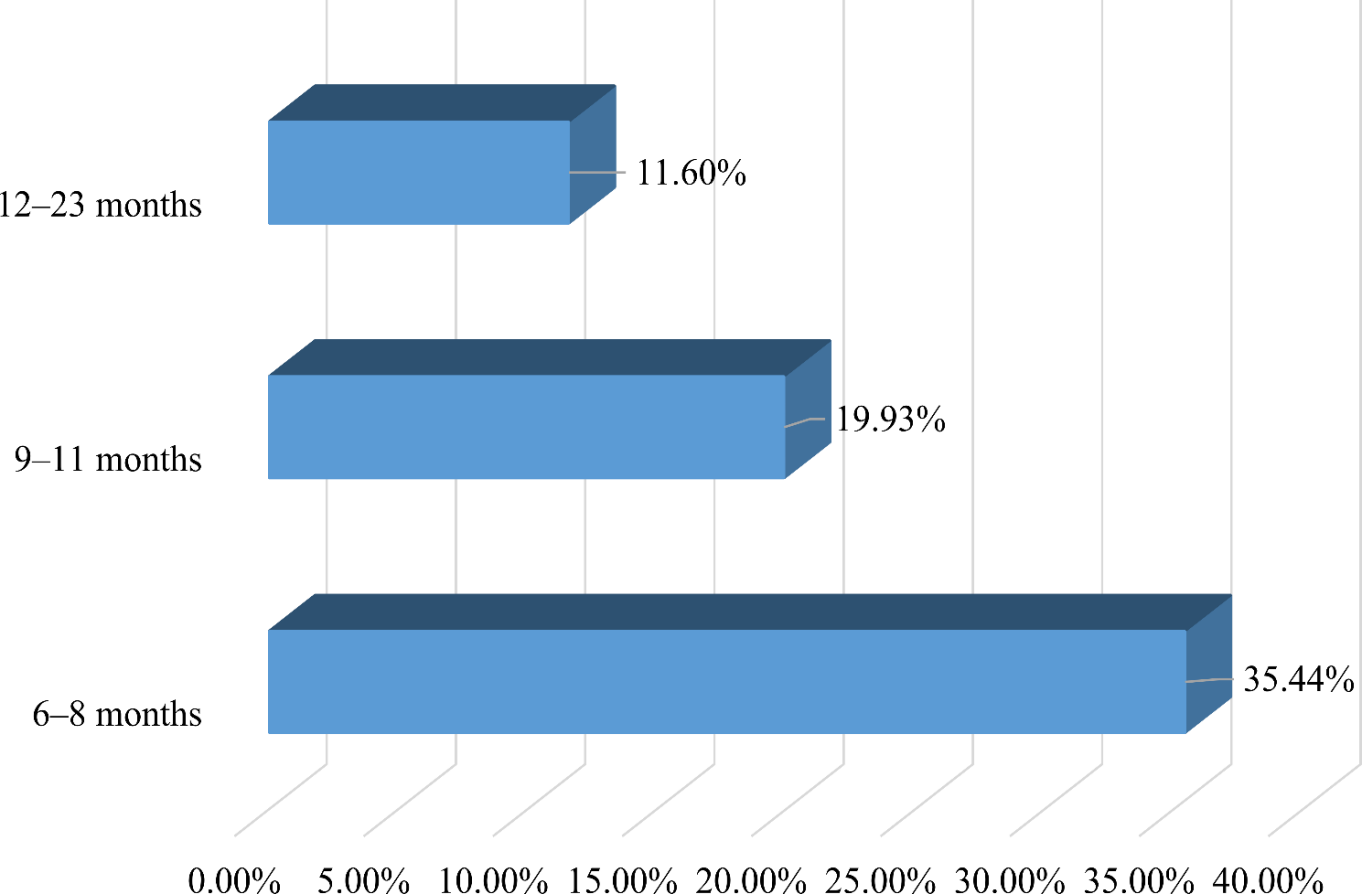
Prevalence of prolonged exclusive breastfeeding by age of the child among children aged 6–23 months in sub-Saharan African countries, DHS 2015–2022

In the present study, the pooled prevalence of prolonged exclusive breastfeeding among children aged 6–23 months in SSA was 17.32% (95% CI: 17.03%, 17.62%). The highest prevalence of PEB was reported in Rwanda (45.89%) and the lowest in Zimbabwe (5.59%) (Fig. 1). The proportion of PEB was also varied by age of the child, in which the highest prevalence was reported among children aged 6–8 months (35.44%), and the lowest among those aged 12–23 months (11.60%) (Fig. 2).

### Measures of variation and model fitness

A null model was used to determine whether the data supported the decision to assess randomness at the community level. Findings from the null model showed that there were significant differences in PEB between communities, with a variance of 0.058 and a P value of < 0.001. The variance within clusters contributed 82.66% of the variation in PEB, while the variance across clusters was responsible for 17.34% of the variation. In the null model, the odds of PEB differed between higher- and lower-risk clusters by a factor of 1.26 times. The intra-class correlation value for Model I indicated that 19.64% of the variation in PEB accounts for the disparities between communities. Then, with the null model, we used community-level variables to generate Model II. According to the ICC value from Model II, cluster variations were the basis for 16.67% of the differences in PEB. In the final model (model III), which attributed approximately 19.23% of the variation in the likelihood of PEB to both individual and community-level variables, the likelihood of PEB varied by 1.28 times across low and high PEB (Table 3).

**Table 3:** Model comparison and random effect analysis for prolonged exclusive breastfeeding and its associated factors in sub-saharan African countries, DHS 2015–2022

Parameter	Null model	Model I	Model II	Model III
Variance	0.0580544	0.0659037	0.055774	0.0644946
ICC	17.34%	19.64%	16.67%	19.23%
MOR	1.26	1.29	1.25	1.28
PCV	Reference	13.52%	3.92%	11.09%
Model fitness				
LLR	-29068.002	-26701.299	-28990.122	-26691.198
Deviance	58,136.004	53,402.598	57,980.244	53,382.396

ICC: Intra cluster correlation, LLR: log-likelihood ratio, MOR: median odds ratio, PCV: Proportional change in variance

### Multilevel analysis of factors associated with prolonged exclusive breastfeeding

In the final fitted model of multivariable multilevel logistic regression (model III), child age, wealth index, maternal educational level, marital status of the mother, media exposure, place of delivery, postnatal checkup, drinking water source, sanitation facility, ANC visits attended during pregnancy, community literacy, and community media exposure were significantly associated with PEB among children aged 6–23 months.

The odds of PEB were 4.39 and 1.97 times higher among children aged 6–8 months and 9–11 months than children aged 12–23 months, respectively [AOR = 4.39; 95% CI (4.17, 4.62)] and [AOR = 1.97; 95% CI (1.86, 2.09)]. Mothers with poor and middle wealth status were 1.15 and 1.08 times more likely to have PEB compared with mothers with rich wealth status, respectively [AOR = 1.15; 95% CI (1.07, 1.23)] and [AOR = 1.08; 95% CI (1.01, 1.16)]. Mothers who had no formal education and completed primary education were 1.56 and 1.22 times more likely to have PEB than mothers with higher education, respectively [AOR = 1.56; 95% CI (1.36, 1.78)] and [AOR = 1.22; 95% CI (1.06, 1.39)]. Unmarried women were 1.11 times more likely to have PEB compared with their counterparts [AOR = 1.11; 95% CI (1.04, 1.19)]. Mothers who had no media exposure were 1.11 times more likely to practice PEB than mothers with media exposure [AOR = 1.11; 95% CI (1.06, 1.17)].

Mothers who gave birth at a health facility were 18% less likely to have PEB compared with those who gave birth at home [AOR = 0.82; 95% CI (0.78, 0.87)]. The odds of PEB were 1.43 times higher among mothers who had no postnatal checkup than their counterparts [AOR = 1.43; 95% CI (1.36, 1.51)]. Mothers with an unimproved drinking water source were 1.06 times more likely to have PEB compared with those with an improved water source [AOR = 1.06; 95% CI (1.01, 1.11)]. The odds of PEB were 1.15 times higher among women with unimproved sanitation facilities than those with improved sanitation facilities [AOR = 1.15; 95% CI (1.10, 1.21)]. Mothers who attended less than four ANC visits during pregnancy were 1.27 times more likely to practice PEB compared with mothers who attended eight or more ANC visits [AOR = 1.27; 95% CI (1.16, 1.39)]. Mothers from communities with low levels of literacy were 1.08 times more likely to have PEB than their counterparts [AOR = 1.08; 95% CI (1.02, 1.15)]. Likewise, mothers from communities with low levels of media exposure were 1.06 times more likely to practice PEB compared with those from communities with high levels of media exposure [AOR = 1.06; 95% CI (1.01, 1.13)] (Table 4).

**Table 4:** Multivariable multilevel logistic regression analysis of individual- and community-level factors associated with prolonged exclusive breastfeeding in SSA, DHS 2015–2022

Variables	Category	Model I AOR (95% CI)	Model II AOR (95% CI)	Model III AOR (95% CI)
Child age	6–8 months	4.39 (4.17, 4.62)*		4.39 (4.17, 4.62)*
	9–11 months	1.97 (1.86, 2.09)*		1.97 (1.86, 2.09)*
	12–23 months	1.00		1.00
Child sex	Male	1.00		1.00
	Female	0.97 (0.93, 1.01)		0.97 (0.93, 1.01)
Household wealth	Poor	1.11 (1.04, 1.18)*		1.15 (1.07, 1.23)*
	Middle	1.06 (0.99, 1.13)		1.08 (1.01, 1.16)*
	Rich	1.00		1.00
Maternal education	No education	1.56 (1.36, 1.79)*		1.56 (1.36, 1.78)*
	Primary	1.21 (1.06, 1.38)*		1.22 (1.06, 1.39)*
	Secondary	1.06 (0.93, 1.21)		1.07 (0.93, 1.22)
	Higher	1.00		1.00
Maternal age	15–24 years	0.98 (0.92, 1.05)		0.98 (0.92, 1.04)
	25–34 years	1.02 (0.97, 1.09)		1.02 (0.97, 1.08)
	35–49 years	1.00		1.00
Marital status	Unmarried	1.12 (1.05, 1.19)*		1.11 (1.04, 1.19)*
	Married	1.00		1.00
Media exposure	No	1.12 (1.06, 1.17)*		1.11 (1.06, 1.17)*
	Yes	1.00		1.00
Place of delivery	Home	1.00		1.00
	Health facility	0.82 (0.78, 0.87)*		0.82 (0.78, 0.87)*
Postnatal checkup	No	1.43 (1.36, 1.51)*		1.43 (1.36, 1.51)*
	Yes	1.00		1.00
Breastfeeding initiation	≥ 1 h of birth	0.92 (0.87, 0.96)*		0.91 (0.87, 1.01)
	< 1 h of birth	1.00		1.00
Drinking water source	Unimproved	1.06 (1.01, 1.11)*		1.06 (1.01, 1.11)*
	Improved	1.00		1.00
Sanitation facility	Unimproved	1.15 (1.09, 1.21)*		1.15 (1.10, 1.21)*
	Improved	1.00		1.00
Antenatal care visits	< 4	1.27 (1.16, 1.39)*		1.27 (1.16, 1.39)*
	4–7	1.04 (0.94, 1.14)		1.04 (0.94, 1.14)
	≥ 8	1.00		1.00
Place of residence	Urban		1.00	1.00
	Rural		1.24 (1.18, 1.30)*	0.93 (0.87, 1.02)
Community literacy	Low		1.16 (1.10, 1.23)*	1.08 (1.02, 1.15)*
	High		1.00	1.00
Community poverty level	Low		1.00	1.00
	High		0.97 (0.91, 1.02)	0.93 (0.88, 1.01)
Community media exposure	Low		1.13 (1.07, 1.20)*	1.06 (1.01, 1.13)*
	High		1.00	1.00

*Statistically significant at p-value < 0.05

## Discussion

This cross-sectional study, with the objective of assessing the pooled prevalence and associated factors of PEB, was conducted in SSA using the recent DHS datasets. The result of this study revealed that the pooled prevalence of PEB was 17.32% (95% CI: 17.03%, 17.62%). This finding was higher than a study conducted in India (7.7%) [28]. On the other hand, the current finding was lower than a study conducted in Bangladesh (29%) [29]. The reason for this could be attributed to the substantial sample size and the inclusion of individuals from many nations with varying socioeconomic backgrounds and cultural customs. Furthermore, the potential explanation for this discrepancy could stem from variations in the participants’ sociodemographic attributes. Policymakers should be on the lookout for promotional biases and stress the significance of nursing exclusively for the first six months of a child’s life. After six months of age, it is also critical to encourage the gradual introduction of complementary foods in order to prevent the incidence of excessively exclusive breastfeeding.

The present study also identified individual and community-level factors associated with PEB. Accordingly, the odds of PEB were higher among children aged 6–8 months and 9–11 months than children aged 12–23 months. This finding was in agreement with a study conducted in Tanzania [30]. Mothers’ misconception that young children cannot digest foods like meat and eggs may be the cause of this. Mothers may also believe that younger infants don’t require a variety of diets or that foods derived from animals may not pass through their digestive systems [31]. Furthermore, a few women were unable to start complementary feeding their babies at six months. Mothers with poor and middle-income status were more likely to have PEB compared with mothers with high income. This finding was consistent with studies conducted in Tanzania [30] and India [12, 28]. This could be because having enough money to purchase essential foods is one of the requirements for the timely initiation of complementary feeding. A previous study also reported that household socioeconomic status (wealth index, food security status, and household income) was a predictor of complementary feeding practices indicators [31].

Mothers who had no formal education and completed primary education were more likely to have PEB than mothers with higher education. Similarly, mothers from communities with low levels of literacy were more likely to have PEB than their counterparts. This finding was in line with studies conducted in Ethiopia [32], Tanzania [30], and India [28]. This can be because maternal education will make them more aware of the advantages of using the best feeding habits for their children. The more education they receive, the more knowledgeable they will be about the value of timely initiation of complementary feeding. Unmarried women were more likely to have PEB compared with their counterparts. This is due to the fact that a husband’s participation in his children’s feeding can have a big impact on the timely initiation of complementary feeding. Mothers who had no media exposure were more likely to practice PEB than mothers with media exposure. Likewise, mothers from communities with low levels of media exposure were more likely to practice PEB compared with those from communities with high levels of media exposure. This finding was in agreement with studies conducted in Ethiopia [33] and Tanzania [30]. The media’s messages are more likely to be embraced because they are typically regarded as reliable sources of information about diet and health [34]. As a result, mothers who had media exposure may learn vital information on the duration of exclusive breastfeeding and the timely initiation of complementary feeding.

Mothers who gave birth at a health facility were less likely to have PEB compared with those who gave birth at home. This finding was consistent with studies conducted in Ethiopia [35–38]. This could be because women who give birth at home might not have received adequate advice on when to begin supplementary feedings. Similarly, mothers who give birth at home would not know enough about the best ways to nurse their children. The odds of PEB were higher among mothers who had no postnatal checkup than their counterparts. This finding was in line with studies conducted in Ethiopia [33, 35, 39] and Tanzania [30]. This may be because women have been receiving education and advice about complementary feeding practices from health experts as part of postnatal care services. The promotion of the prompt commencement of supplemental feeding is positively impacted by mothers who receive health education and advice regarding the practice during postnatal care.

Mothers with an unimproved drinking water source and sanitation facility were more likely to have PEB compared with their counterparts. Adequate complementary feeding practice necessitates adequate food availability at the household level, proper nutritional knowledge application by caregivers, and hygienic preparation of complementary foods [40]. Increased risks of cholera, typhoid, schistosomiasis, and infections of the skin, eyes, and respiratory systems are caused by unimproved sanitation facilities and sources of drinking water [41, 42], which interferes with the timely initiation of complementary feeding. Mothers who attended less than four ANC visits during pregnancy were more likely to practice PEB compared with mothers who attended eight or more ANC visits. This finding was in line with studies conducted in Ethiopia [33, 43]. Counseling on self-feeding and nursing an infant is one of the services provided during antenatal care. Mothers are even advised to cook a variety of foods for their kids. Thus, the greater the number of antenatal care visits, the greater the number of mothers who receive counseling services and subsequently use them.

### Strengths and limitations of the study

The following are the strengths of the current study: First, weighted nationally representative data from 21 SSA countries was used to create a large sample size. Second, in order to account for the hierarchical structure of the DHS data and obtain a trustworthy estimate, a multilevel analysis was used. Third, as this study uses pooled nationwide survey data, policymakers and program administrators could use its findings as input to create suitable intervention methods to enhance child health. Furthermore, this investigation had the following limitations: Firstly, because the DHS survey relied on mothers’ self-reports, social desirability and recall biases may have affected the study’s findings. Secondly, because the data were cross-sectional in nature, it was unable to determine the cause-and-effect relationship between the variables. In addition, some variables, like knowledge and attitude of mothers about PEB, were not included due to the secondary nature of the data.

## Conclusions

Nearly one out of five children aged 6–23 months in sub-Saharan Africa had prolonged exclusive breastfeeding. Both individual- and community-level factors were significantly associated with prolonged exclusive breastfeeding. Policymakers could find it very important to support maternal education, poverty reduction, media exposure, maternal healthcare services, and complementary feeding hygiene practices in order to encourage the timely initiation of complementary feeding. Timely introduction of complementary feeding is essential to reduce nutritional deficiencies, including those of micronutrients like iron, electrolytes, vitamin B12, and others, and the clinical adverse outcomes related to them.

## Data Availability

The data from the 21 SSA countries is publicly available online at https://dhsprogram.com/data/available-datasets.cfm.

## Abbreviations

ANC: Antenatal Care
AOR: Adjusted Odds Ratio
CI: Confidence Interval
DHS: Demographic and Health Survey
ICC: Intra-class Correlation Coefficient
LMICs: low- and middle-income countries
MOR: Median Odds Ratio
PCV: Proportional Change in Variance
PEB: Prolonged Exclusive Breastfeeding
PNC: Postnatal Care
SSA: sub-Saharan Africa
WHO: World Health Organization
